# Enrichment of novel CD3+F4/80+ cells in brown adipose tissue following adrenergic stimulation

**DOI:** 10.3389/fimmu.2024.1455407

**Published:** 2024-08-27

**Authors:** Hee-Don Chae, Jelena Levi

**Affiliations:** CellSight Technologies Incorporated, San Francisco, CA, United States

**Keywords:** macrophage, dual lineage, brown adipose tissue, thermogenesis, sympathetic nerve

## Abstract

Macrophages play a multifaceted role in maintaining tissue homeostasis, fighting infections, and regulating cold-induced thermogenesis. The brown adipose tissue (BAT) is crucial for maintaining body temperature during cold exposure. Cold stress triggers the sympathetic nervous system to release norepinephrine (NE), which activates BAT via β3-adrenergic receptors, initiating lipolysis and glycolysis. BAT-infiltrating macrophages can either hinder or enhance thermogenesis by controlling the interplay between BAT cells and sympathetic nerves. In this study we report on a unique population of CD3+F4/80+ dual lineage co-expressing (DE) cells within the interscapular BAT (iBAT), that increased following chronic adrenergic stimulation. In forward scatter/side scatter plots, they formed a cluster distinct from lymphocytes, appearing larger and more complex. These CD3+F4/80+ DE cells demonstrated the lack of T cell markers CD62L and TCRβ and expressed higher levels of Ly6C, F4/80, and CD11b markers compared to T cells and CD3- macrophages. Furthermore, analysis revealed two subpopulations within the CD3+F4/80+ DE population based on MHCII expression, with the proportion of MHCII-low subset increasing with adrenergic stimulation. This novel DE population within iBAT, unequivocally identified by the its unique surface marker profile, warrants further investigation into the intricate mechanisms governing adaptive thermogenesis regulation.

## Introduction

Adipose tissue (AT), primarily composed of adipocytes (fat cells), serves as a dynamic immune and endocrine organ, while also playing essential roles in controlling energy and glucose homeostasis. Within AT, a diverse array of cell populations, including connective tissues, stromal vascular cells, endothelial cells, nerve tissue, lymphocytes, and other inflammatory cells, contribute to its various immunological and physiological functions (1–4). White adipose tissue (WAT), distributed throughout the body, predominantly functions in energy storage. Conversely, brown adipose tissue (BAT), found in specific highly vascularized and innervated deposits like the interscapular region, is specialized for energy dissipation through non-shivering heat production. This thermogenic process is vital for maintaining body temperature homeostasis (1, 5). BAT adipocytes are characterized by small multilocular lipid droplets and a higher abundance of cristae-dense, fragmented mitochondria expressing uncoupling protein 1 (UCP1). These features enable increased rates of lipolysis and oxidative energy production. UCP1 functions by uncoupling respiration from ATP synthesis, dissipating the proton gradient across the inner mitochondrial membrane and generating heat. Cold exposure stimulates the thermogenic activity of BAT by triggering the release of norepinephrine (NE) from sympathetic nerve terminals. NE activates BAT thermogenesis via the β3-adrenergic receptor on adipocytes, thereby promoting lipolysis and UCP1 activation (3, 6, 7).

BAT harbors a diverse spectrum of immune cells, including macrophages, monocytes, neutrophils, eosinophils, dendritic cells, mast cells, T cells, B cells, natural killer cells, and innate lymphoid cells (3, 5). These immune cells play an active role in regulating the thermogenic activity of BAT, and metabolic stress has been shown to alter the immune landscape within BAT (5, 8–10). Prolonged cold exposure-induced BAT adipogenesis is associated with the recruitment of myeloid cells, particularly macrophages, into BAT (3, 9, 10).

Macrophages, crucial tissue-infiltrating immune cells, play pivotal roles in innate immunity, inflammation, homeostasis, tissue repair and remodeling, clearance of cellular debris, and metabolic regulation owing to their heterogeneity, plasticity, and polarization (11–13). In obesity, macrophages become the predominant infiltrating cells in WAT, driving inflammation and influencing systemic energy homeostasis (14). Macrophages constitute a major portion of the immune cells in BAT that accumulate during BAT expansion by cold exposure (7, 9, 15). Recent findings indicate that macrophages regulate the thermogenic activity of BAT through various mechanisms (7, 16). The accumulation of pro-inflammatory M1 macrophages, for instance, suppresses the induction of thermogenic adipocytes in obese adipose tissues via TNFα (17). CD206-positive BAT macrophages were found to eliminate damaged mitochondria during cold exposure, ensuring optimal BAT thermogenesis (15). Interestingly, macrophages have been shown to control thermogenesis by modulating the interaction between BAT cells and sympathetic nerves (18–21). The roles of macrophages in controlling the sympathetic nervous system and β3- adrenergic receptor activation are complex and context-dependent. Macrophages expressing solute carrier family 6 member 2 (Slc6a2) play an inhibitory role in thermogenesis by eliminating NE through the NE transporter Slc6a2 and the NE-degrading enzyme monoamine oxidase A (18, 20). On the other hand, macrophages expressing methyl-CpG-binding protein 2 are critical for the local NE signaling, supporting optimal UCP1 expression and BAT thermogenesis (21). Since BAT activation triggers dramatic changes in the composition of its macrophage subsets (9, 15, 22), understanding the phenotypes and functions of these distinct populations is essential to elucidate the regulatory mechanisms of adaptive thermogenesis. To this end, we performed in-depth immunophenotyping of BAT-infiltrating immune cells following the prolonged activation of β3-adrenergic receptor.

In this study, we identified a novel CD3+F4/80+ dual lineage co-expressing (DE) population in the interscapular BAT (iBAT) using multi-parameter flow cytometry. Notably, CD3+F4/80+ DE cells were found to be enriched following prolonged adrenergic stimulation.

## Materials and methods

### Cell preparation

C57BL/6J mice, aged 6 to 9 weeks, were procured from The Jackson Laboratories in Bar Harbor, ME. The ß3-adrenergic receptor agonist BRL37344 (Tocris Bioscience, Minneapolis, MN) or PBS was administered intraperitoneally to mice at a dose of 10 mg/kg body weight for four consecutive days. Two hours after the last administration, the animals were euthanized. Murine experiments were reviewed and approved by the University of California, San Francisco Institutional Animal Care and Use Committee. For preparing cells from iBAT, the tissue was finely minced with scissors in M199 buffer (M199 media from Life Technologies, Grand Island, NY, containing 2% BSA and 2.5 mM glucose). Subsequently, the chopped iBAT samples were subjected to digestion with 1 mg/ml collagenase D (Sigma, St. Louis, MO) and 20 U/ml DNase I (Sigma) at 37°C in a shaking incubator for 30 minutes. The digested tissue was then filtered through a 150-μm cell strainer, and the resulting cell suspension was centrifuged at 350 × g for 5 minutes. The pelleted stromal vascular fraction (SVF) cells were treated with red blood cell lysis buffer (Invitrogen, Eugene, OR). Bone marrow (BM) cells were collected from the femur. Whole BM was flushed by centrifugation at 3,000g for 1 minute. The pellet was suspended in red blood cell lysis buffer to remove red blood cells for 1 minute at room temperature. Cells were washed again with M199 buffer.

### Flow cytometry

Single-cell suspensions were Fc-receptor blocked with Fc-receptor blocker (BioLegend, San Diego, CA) before staining with fluorochrome-conjugated antibodies. The following fluorochrome-conjugated antibodies were used: Alexa Fluor^®^ 488 anti-mouse CD3ϵ (#100321), Brilliant Violet 510™ anti-mouse CD4 (#100449), PerCP/Cyanine5.5 anti-mouse CD8a (#100734), PerCP/Cyanine5.5 anti-mouse CD9 (#124818), Brilliant Violet 711™ anti-mouse CD11c (#117349), Brilliant Violet 785™ anti-mouse CD25 (#102051), APC anti-mouse CD31 (#102410), PE/Cyanine7 anti-mouse CD34 (#119326), Alexa Fluor^®^ 700 anti-mouse CD45.2 (#109822), PE anti-mouse CD69 (#104508), PE/Cyanine7 anti-mouse CD127 (IL-7Rα) (#135014), PE/Dazzle™ 594 anti-mouse F4/80 ($123156), Brilliant Violet 650™ anti-mouse Ly-6C (#128049), PE anti-mouse Ly-6G (#127607), Brilliant Violet 605™ anti-mouse NK-1.1 (#108753), Brilliant Violet 650™ anti-mouse NK-1.1 (#108736), Brilliant Violet 605™ anti-mouse CD279 (PD-1) (#135220), Brilliant Violet 711™ anti-mouse TCR β chain (#109243, Biolegend). Cells were stained with DAPI (100ng/ml, Sigma) to exclude dead cells. Samples were acquired with Attune NxT flow cytometer (Invitrogen, Carlsbad, CA). Flow cytometry data were analyzed using FlowJo software (v10.01, TreeStar, Ashland, OR, USA). t-distributed stochastic neighbor embedding (tSNE) plots were generated with default FlowJo setting.

### Statistical analysis

All data were reported as the mean ± standard deviation (SD). P values for statistical significance were obtained by using an unpaired Student t test or one-way ANOVA using Prism software (GraphPad Software, La Jolla, CA, USA). P ≤ 0.05 was considered significant. In the figures, asterisks denote statistical significance (* P ≤ 0.05, ** P ≤ 0.01, *** P ≤ 0.001, **** P ≤ 0.0001).

## Results

### Enrichment of novel CD3+F4/80+ DE population in adrenergically stimulated iBAT

**Figure 1:**
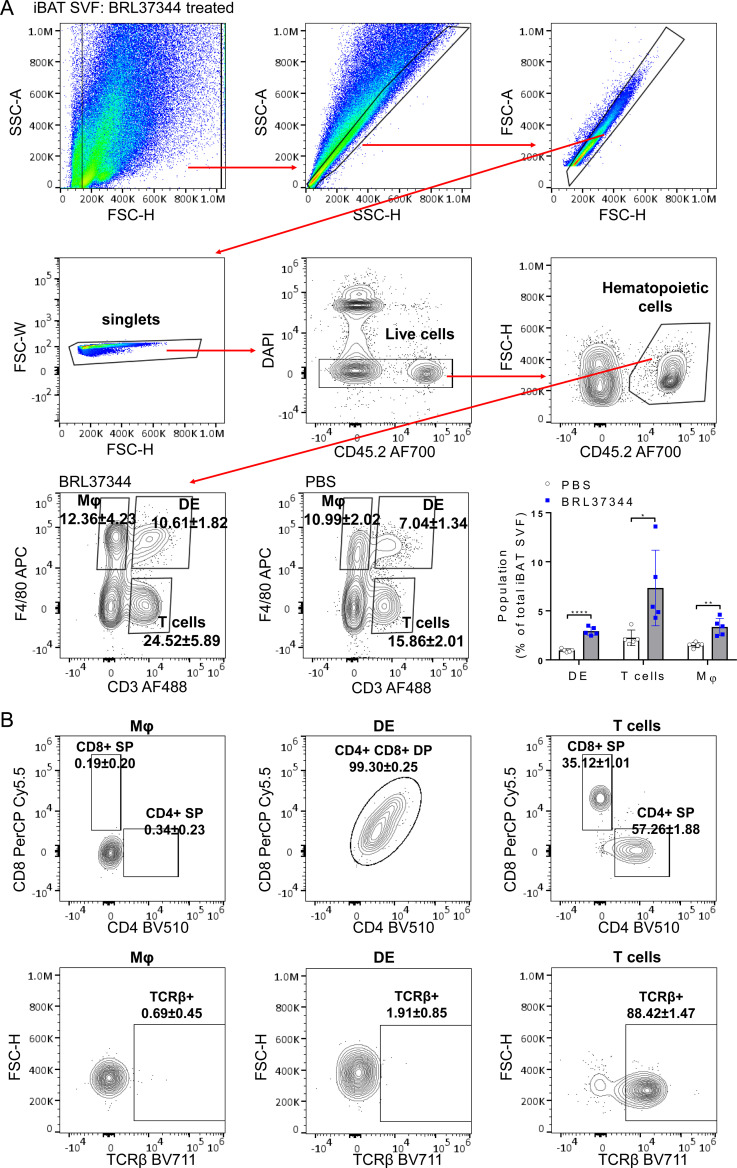
Enrichment of CD3+F4/80+ DE cells in adrenergically stimulated iBAT SVF. iBAT SVF were collected from mice treated with the β3-adrenergic agonist BRL37344 (10mg/kg) for 4 days. **(A)** Gating strategy to distinguish CD3+F4/80+ DE cells from T cells (CD3+ F4/80-) and macrophages (Mφ) (CD3-F4/80+) in iBAT SVF. Single, live, CD45+ hematopoietic cells were gated, and CD3+F4/80+ DE, CD3+ F4/80- T cells, and CD3-F4/80+ Mφ were identified. Representative FACS density plots indicate proportions of CD3+F4/80+ DE, CD3+ F4/80- T cells, and CD3-F4/80+ Mφ within CD45+ hematopoietic cells. The frequencies of T cells, macrophages, and DE cells are plotted as a percentage of the total live iBAT SVF cells. **(B)** Characterization of T cells, macrophages, and CD3+F4/80+ DE cells based on the surface expression of CD4, CD8 (top panels), and TCRβ (bottom panels). CD3+F4/80+ DE cells expressed both CD4 and CD8 but lacked TCRβ expression. Each data point represents an individual mouse. Data are presented as mean ± SD (n = 5). * P ≤ 0.05, ** P ≤ 0.01, **** P ≤ 0.0001.

We explored changes in the immune landscape of iBAT that occur after prolonged adrenergic stimulation using multi-parameter flow cytometry. Notably, we identified a previously unreported CD3+F4/80+ DE cells among live CD45-positive iBAT SVF hematopoietic cells. Along with percentages of CD3-F4/80+ macrophages and CD3+F4/80- T cells, the frequency of CD3+F4/80+ subset in iBAT SVF increased approximately threefold after adrenergic stimulation (**Figure 1A**). Our analysis extended to examination of the expression of surface T cell markers (TCRβ, CD4, and CD8) on CD3+F4/80+ cells. While CD3+F4/80- T cells were identified as TCRβ+CD4+ or CD8+ single positive (SP) T cells, CD3+F4/80+ DE population was characterized as TCRβ-CD4+CD8+ cells (**Figure 1B**). Given that regulated BM adipocytes respond to various physiological conditions in the red marrow, including β-adrenergic stimulation (23, 24), we also investigated the presence of CD3+F4/80+ DE cells in the femur BM. The BM also harbored a population of CD3+F4/80+ DE cells, but both this population and the CD3-F4/80+ macrophage population declined with β-adrenergic stimulation (**Supplementary Figure 1A**). Notably, only one third of the BM CD3+F4/80+ population consisted of TCRβ-CD4+CD8+ cells, exhibiting the highest F4/80 surface expression (**Supplementary Figure 1B**). Interestingly, CD3+F4/80+TCRβ-CD4+CD8+ subpopulation decreased following β-adrenergic stimulation, whereas CD3+F4/80+TCRβ+ cells did not (**Supplementary Figure 1C**).

### The CD3+F4/80+ DE population exhibits a distinct flow cytometry profile compared to T cells

**Figure 2:**
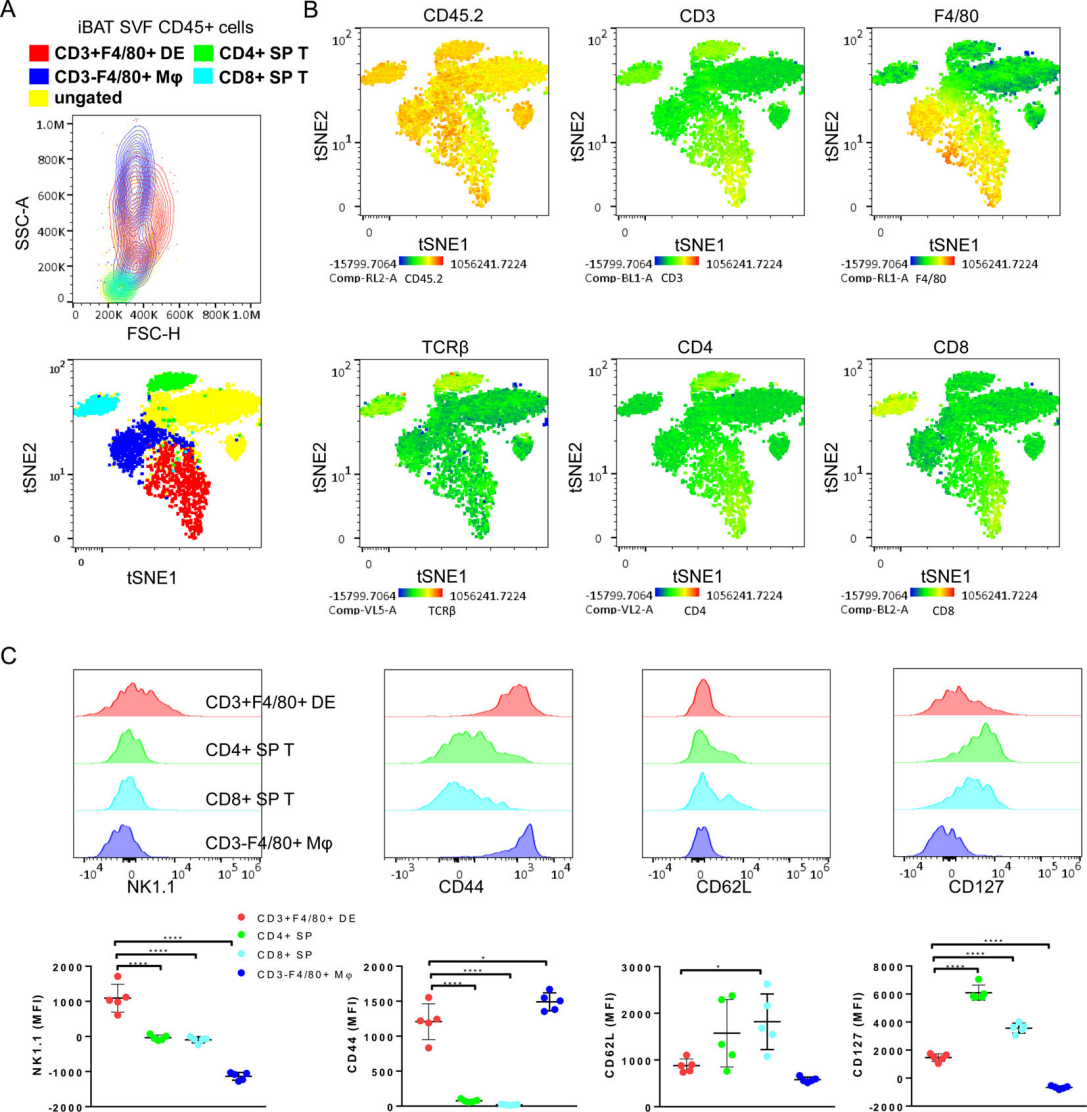
Defining immunophenotype of CD3+F4/80+ DE cells with T/NK cells-related markers. **(A)** A representative FSC/SSC plot (top) and tSNE flow cytometry plot (bottom) displaying the color-coded subsets for CD45+ subsets of iBAT SVF from BRL37344-treated mice. The gating strategy for CD45+ subsets in iBAT SVF is described in **Figure 1**. The ungated population (yellow) remains unidentified by these definitions. CD45+ subsets were overlaid onto a tSNE plot, and clustered on CD45+ cells of adrenergically stimulated iBAT SVF. The tSNE flow cytometry plot was based on the surface expression of markers including CD45, CD3, CD4, CD8, CD62L, CD44, CD25, CD127, CD69, PD1, NK1.1, and F4/80. **(B)** Color scaling of the tSNE plots visualizing the relative surface expression levels of CD45.2, CD3, CD4, CD8, TCRβ and F4/80 in iBAT SVF CD45+ cells. **(C)** Expression levels of T cell or NK cell-associated markers in CD3+F4/80+ DE cells (red), CD4+ SP T cells (green), CD8+ SP T cells (cyan), and macrophages (blue). Representative histograms (top panels) and quantification of surface marker expression (measured as MFI) for each population. Each data point represents an individual mouse. Data are shown as mean ± SD (n = 5). * P ≤ 0.05, **** P ≤ 0.0001.

The iBAT CD3+F4/80+ DE cells were verified to be positioned within the size and granularity characteristics of monocytes/macrophages/granulocytes (Top plot, **Figure 2A**). Macrophages lacking CD3 expression exhibited the formation of two distinct clusters in the forward scatter and side scatter plot. Within these, CD3+F4/80+ DE cells clustered, displaying bigger and more granular cellular structures compared to lymphocytes (Top plot, **Figure 2A**). To confirm that CD3+F4/80+ DE cells constitute a distinct population separate from normal T cells and macrophages, we further examined the expression of surface markers characteristic of T/NK cells on CD3+F4/80+ DE cells. Multidimensional flow cytometry analysis of CD45+ iBAT SVF hematopoietic cells was performed, and subsets of CD3+F4/80+ DE cells, CD4+ T cells, CD8+ T cells, and CD3- macrophages were visualized in a tSNE plot with a set of 12 T/NK cell-associated markers (CD45, CD3, CD4, CD8, CD62L, CD44, CD25, CD127, CD69, PD1, NK1.1, and F4/80), demonstrating distinct clusters in a tSNE plot (Bottom plot, **Figure 2A**). The CD3+F4/80+ subset was characterized by positive expression of CD45.2, CD3, F4/80, CD4, CD8, and the absence of TCRβ, as shown in tSNE plots overlaid with heatmaps of each marker (**Figure 2B**). This finding was consistent with two-dimensional dot plot analysis (**Figures 1A, B**). Further supporting their distinct identity, CD3+F4/80+ DE cells displayed expression levels of CD45.2, a ubiquitous pan-leukocyte marker, comparable to T cells and CD3- macrophages (**Figure 2B**). The absence of significantly elevated CD45.2 expression in CD3+F4/80+ DE cells disfavors the hypothesis that they represent mere aggregates of T cells and macrophages. Population overlays revealed differential marker expression, with higher NK1.1 median fluorescence intensity (MFI) in CD3+F4/80+ DE cells compared to T cells and CD3- macrophages. Notably, CD3+F4/80+ DE cells exhibited a significant decrease in CD127 and CD62L expression compared to T cells. The MFI for CD44 was increased in CD3+F4/80+ DE cells compared to T cells, but slightly lower than in CD3- macrophages (**Figure 2C**). CD3+F4/80+ DE cells did not express CD25, PD1, and CD69.

### CD3+F4/80+ DE cells display a unique expression pattern of myeloid cell markers

**Figure 3:**
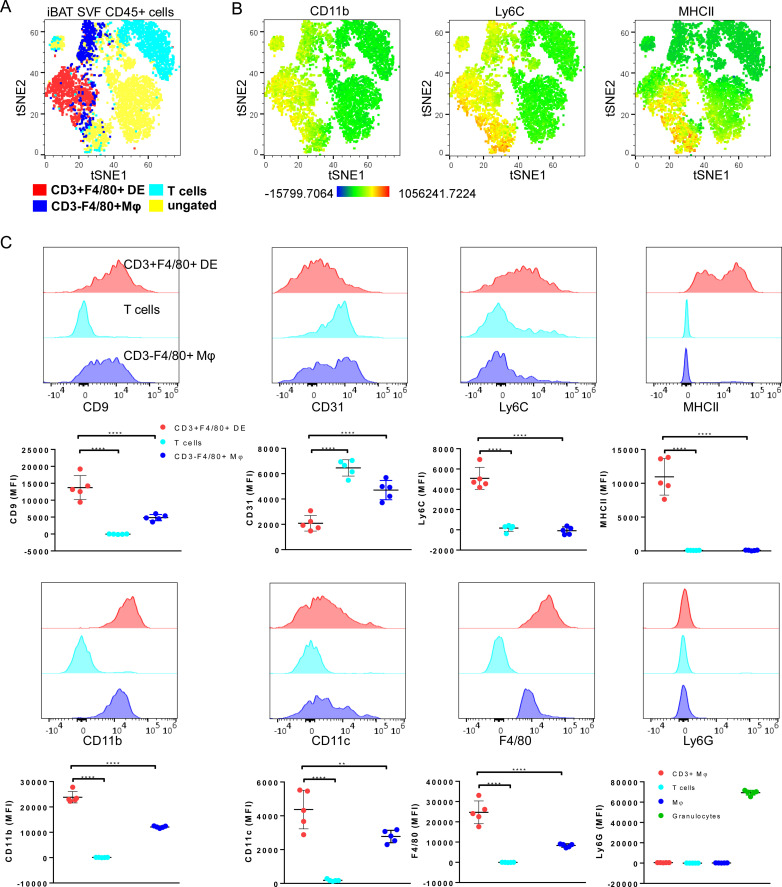
Further characterization of CD3+F4/80+ DE cells with myeloid cell-associated markers. **(A)** A tSNE plot annotated with CD3+F4/80+ DE cells, T cells (CD3+ F4/80-), and macrophages (CD3- F4/80+) of adrenergically stimulated iBAT SVF, demonstrating that CD3+F4/80+ DE cells do not fall into the lymphocytes and macrophage populations. Clustering was performed using 12 markers including CD45, CD3, F4/80, CD11b, CD11c, CD9, CD31, CD34, Ly6C, Ly6G, MHCII, and NK1.1. **(B)** Color scaling of the tSNE plots illustrating the relative intensity of CD11b, Ly6C, and MHCII in iBAT SVF CD45+ cells. **(C)** Expression of myeloid cell-associated surface markers for CD3+F4/80+ DE cells (red), T cells (cyan), and macrophages (blue) in adrenergically stimulated-iBAT SVF cells. Each data point represents an individual mouse. Data are presented as mean ± SD (n = 5). ** P ≤ 0.01, **** P ≤ 0.0001.

**Figure 4:**
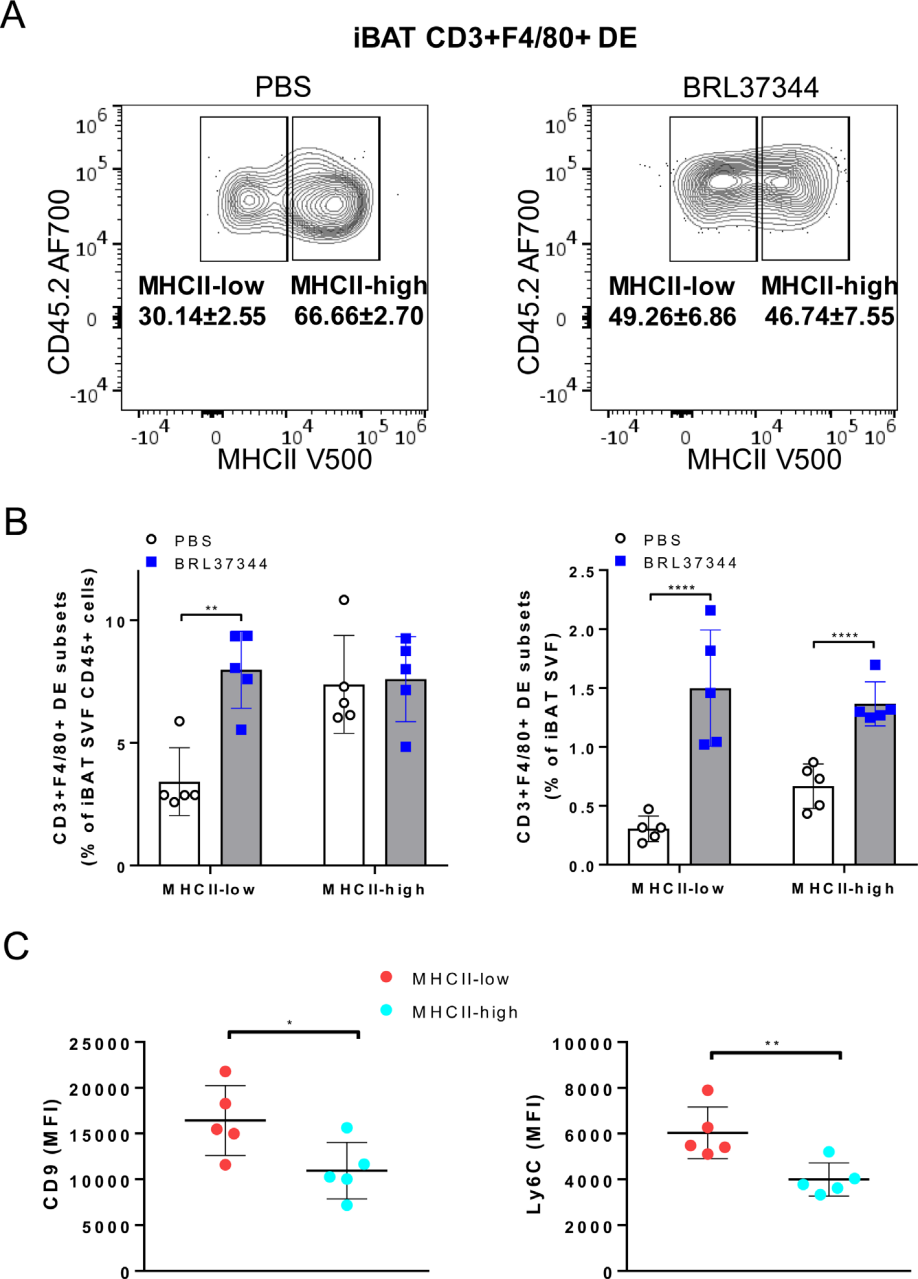
Increase of MHCII-low CD3+F4/80+ DE cells in iBAT following adrenergic stimulation. **(A)** CD3+F4/80+ DE cells from iBAT were plotted onto MHCII/CD45.2 histogram. The proportion of MHCII-low subset in CD3+F4/80+ DE population increased with chronic adrenergic stimulation. **(B)** The frequencies of MHCII-high and MHCII-low CD3+F4/80+ DE cells subsets are depicted as a percentage of CD45+ or total live iBAT SVF cells. **(C)** Expression levels of CD9 and Ly6C in CD3+F4/80+ MHCII-low DE cells (red) and CD3+F4/80+ MHCII-high DE cells (cyan). Quantification of MFI of CD9 and Ly6C expression in CD3+F4/80+ MHCII-low and MHCII-high macrophages. Each data point represents an individual mouse. Data are shown as mean ± SD (n = 5). * P ≤ 0.05, ** P ≤ 0.01, **** P ≤ 0.0001.

To further characterize the CD3+F4/80+ DE subset, we conducted a comprehensive analysis of differential expression involving surface markers commonly associated with myeloid/lymphoid cells (CD45, F4/80, CD11b, CD11c, CD9, CD31, CD34, Ly6C, Ly6G, MHCII, CD3 and NK1.1). tSNE gating overlays and density plots for CD11b, Ly6C, and MHCII revealed distinct segregation of iBAT CD3+F4/80+ DE cells (CD45+CD3+F4/80+CD11b+MHCII+) from T lymphocytes and CD3- macrophages (**Figures 3A, B**). Additionally, CD3+F4/80+ DE cells displayed distinct expression patterns for various markers compared to lymphocytes and macrophages (**Figure 3C**), providing further insight into their unique identity. While CD3+F4/80+ DE cells exhibited increased expression of CD9, Ly6C, MHCII, CD11b, CD11c, and F4/80 compared to lymphocytes and macrophages, the expression level of CD31 in CD3+F4/80+ DE cells was lower than that in lymphocytes and macrophages. Furthermore, they tested negative for Ly6G and CD34. The CD3+F4/80+ DE population in iBAT exhibits further heterogeneity. Analysis revealed two distinct subpopulations based on their MHCII expression levels: MHCII-low and MHCII-high (**Figure 3C**). Considering that MHCII expression in macrophages tends to be downregulated after injury to facilitate tissue repair (25, 26), we investigated whether chronic adrenergic stimulation could influence MHCII expression in CD3+F4/80+ DE cells. Our findings demonstrate a significant increase in the proportion of the MHCII-low subset within the CD3+F4/80+ population following chronic adrenergic stimulation (**Figure 4A**). Both MHCII-high and MHCII-low CD3+F4/80+ DE populations increased in iBAT SVF following adrenergic stimulation. However, while the frequency of MHCII-high CD3+F4/80+ DE cells remained unchanged in the CD45+ iBAT hematopoietic population, the frequency of MHCII-low CD3+F4/80+ DE cells more than doubled (**Figure 4B**). This indicates that the ratio of MHCII-high to MHCII-low CD3+F4/80+ cells shifted towards the MHCII-low phenotype after BRL37344 administration. Notably, the MHCII-low subset displayed a marked upregulation of CD9 and Ly6C markers compared to their MHCII-high counterparts (**Figure 4C**). In summary, our results show that extended adrenergic stimulation significantly increases novel iBAT CD3+F4/80+ DE population, particularly those expressing low levels of MHCII.

## Discussion

Our investigation reveals a novel DE population co-expressing CD3, CD4, CD8, CD11b, F4/80, and lacking TCRβ and Ly6G within iBAT. This unique marker expression profile, encompassing features of both lymphocytes and myeloid cells, distinguishes them from conventional macrophages and T cells, leading us to designate them as a distinct myeloid subset.

Prior studies have identified rare myeloid populations expressing dual markers associated with both lymphocytes and myeloid cells, including CD19+CD11b+ B cells in mouse brain, TCRαβ+CD11b+ mouse macrophages and TCRαβ+CD14+ human macrophages in the tumor microenvironment, and CD3+CD45+CD68+CD11b+C1q+ tumor-associated macrophages, CD3+TCRαβ+CD11b+ and CD3+TCRαβ-CD11b+ macrophages during a *Plasmodium berghei* ANKA infection, CD3+CD19+ cells in human immunodeficiency virus-*Mycobacterium tuberculosis* coinfection, CD3-CD4+CD8+CD11c+CD80+MHCII+CD68+CD163+CD25-CD103-CD49b- monocytes/macrophages in T-cell leukemia virus type-I pX transgenic rats (27–35). Notably, the surface marker profile of these previously reported DE cells differs from that of CD3+F4/80+ population identified within iBAT.

Recently, Burel et al. elucidated that the detection of unexpected subsets expressing lineage markers of distinct populations, such as CD3+CD14+ cells, may be attributed to various factors, including the formation of cell-cell complexes, where T cells are closely associated with debris derived from myeloid cells, or the phenomenon of trogocytosis, wherein T cells acquire membrane fragments from myeloid cells (36, 37). Our data contrasts these observations. CD3+CD14-high cells from latent tuberculosis patients displayed elevated forward scatter/side scatter and CD45 fluorescence, lacked distinct surface markers, and resembled both T cells and monocytes. In contrast, iBAT CD3+F4/80+ DE cells resided within the same FSC/SSC region as CD3-F4/80+ macrophages, exhibited comparable expression level of CD45 and expressed distinct levels of lymphocyte and myeloid markers, differentiating them from classical immune cells. Therefore, our findings suggest that iBAT CD3+F4/80+ DE cells represent a distinctive myeloid population rather than artifacts of cell-cell interactions. The presence of iBAT CD3+F4/80+ cells expressing dual lineage markers has not been reported thus far, primarily because T cell (CD3) lineage marker-positive cells are typically gated out early in the analysis of myeloid populations (36).

Previous research has demonstrated an age-dependent increase in MHCII expression on macrophages within iBAT, heart, and kidney tissues (21, 25, 26). Following injury, MHCII low expressing macrophages become dominant to facilitate debris removal and tissue repair in heart and kidney (25, 26). Notably, MHCII-low macrophages are the primary population following myocardial infarction and exhibit enhanced phagocytic activity and strong anti-inflammatory properties crucial for cardiac tissue repair (25). Cold exposure, while suppressing the F4/80+MHCII-high population, conversely promotes an increase in anti-inflammatory MHCII-dim macrophage subset (9). Our study aligns with these findings, as prolonged adrenergic stimulation in iBAT resulted in an elevated proportion of MHCII-low CD3+F4/80+ DE cells (**Figure 4**). Given that phagocytic macrophages accumulate in BAT during cold exposure to eliminate oxidatively damaged mitochondria-derived extracellular vesicles, thereby maintaining optimal thermogenesis (15), MHCII-low CD3+F4/80+ DE cells likely play a critical role in preserving thermogenic capacity of BAT.

Taken together our results suggest a link between adrenergic stimulation, MHCII downregulation, and the expansion of a specific myeloid subset within BAT. Further investigation into the function of these distinct subpopulations is crucial for understanding their contribution to thermogenesis regulation.

## Data Availability

The original contributions presented in the study are included in the article/**Supplementary Material**. Further inquiries can be directed to the corresponding authors.
