# Supplementary material for: Enrichment of novel CD3+F4/80+ cells in brown adipose tissue following adrenergic stimulation
Source: Front Immunol. 2024 Aug 27;15:1455407. doi: 10.3389/fimmu.2024.1455407 (PMC11384597; doi:10.3389/fimmu.2024.1455407)
Supplement: Supplementary file 1 [file DataSheet1.pdf]

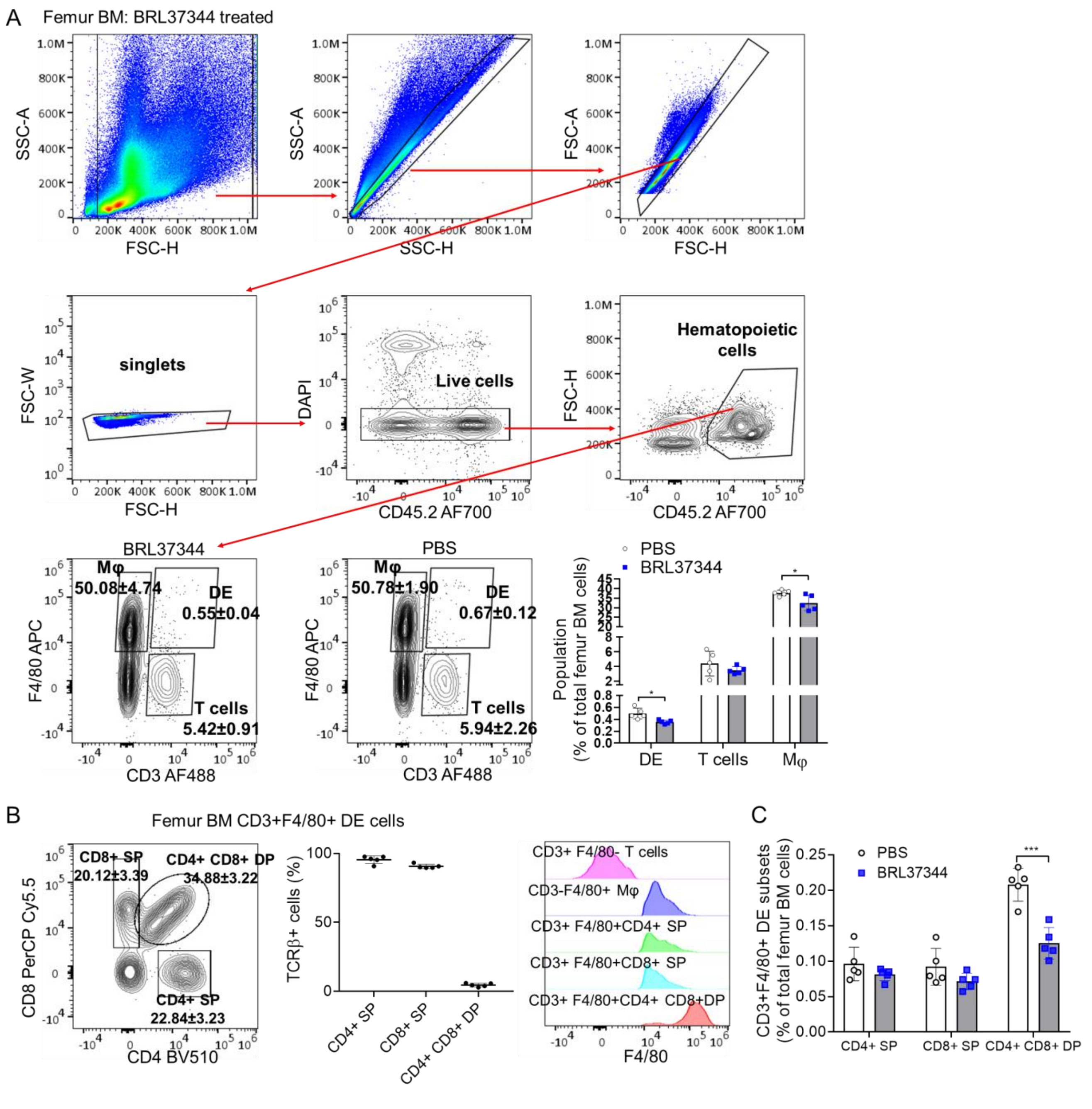

Supplementary Figure 1. CD3+F4/80+ DE cells in femur BM. Femur BM cells were collected from mice treated with the β3-adrenergic agonist BRL37344 (10mg/kg) for 4 days. (A) Gating strategy to identify CD3+F4/80+ DE cells in the femur BM. Single, live, CD45+ hematopoietic cells were gated, and CD3+F4/80+ DE, CD3+ F4/80- T cells, and CD3-F4/80+ macrophages (Mφ) were identified. The proportion of each sub-population within the femur BM CD45+ hematopoietic cells is displayed in the flow cytometry plots. The frequencies of macrophages and DE cells decreased in the femur BM following adrenergic stimulation. The frequencies of T cells, macrophages, and DE cells are plotted as a percentage of the total live femur BM cells. (B) CD4/CD8 double positive (DP) CD3+F4/80+ DE BM cells were negative for TCRβ and exhibited high F4/80 expression. (C) Following adrenergic stimulation, there was a decrease in the proportion of CD4/CD8 DP CD3+F4/80+ DE cells in the femur BM. Each data point represents an individual mouse. Data are presented as mean ± SD (n = 5). * $p \leq 0.05$, *** $p \leq 0.001$.
